# Horizontal gene transfer of microbial cellulases into nematode genomes is associated with functional assimilation and gene turnover

**DOI:** 10.1186/1471-2148-11-13

**Published:** 2011-01-13

**Authors:** Werner E Mayer, Lisa N Schuster, Gabi Bartelmes, Christoph Dieterich, Ralf J Sommer

**Affiliations:** 1 Max-Planck Institute for Developmental Biology, Department for Evolutionary Biology, Spemannstrasse 37, 72076 Tübingen, Germany; 2 Max-Delbrueck-Centre for Molecular Medicine, Berlin

## Abstract

**Background:**

Natural acquisition of novel genes from other organisms by horizontal or lateral gene transfer is well established for microorganisms. There is now growing evidence that horizontal gene transfer also plays important roles in the evolution of eukaryotes. Genome-sequencing and EST projects of plant and animal associated nematodes such as *Brugia*, *Meloidogyne*, *Bursaphelenchus *and *Pristionchus *indicate horizontal gene transfer as a key adaptation towards parasitism and pathogenicity. However, little is known about the functional activity and evolutionary longevity of genes acquired by horizontal gene transfer and the mechanisms favoring such processes.

**Results:**

We examine the transfer of cellulase genes to the free-living and beetle-associated nematode *Pristionchus pacificus*, for which detailed phylogenetic knowledge is available, to address predictions by evolutionary theory for successful gene transfer. We used transcriptomics in seven *Pristionchus *species and three other related diplogastrid nematodes with a well-defined phylogenetic framework to study the evolution of ancestral cellulase genes acquired by horizontal gene transfer. We performed intra-species, inter-species and inter-genic analysis by comparing the transcriptomes of these ten species and tested for cellulase activity in each species. Species with cellulase genes in their transcriptome always exhibited cellulase activity indicating functional integration into the host's genome and biology. The phylogenetic profile of cellulase genes was congruent with the species phylogeny demonstrating gene longevity. Cellulase genes show notable turnover with elevated birth and death rates. Comparison by sequencing of three selected cellulase genes in 24 natural isolates of *Pristionchus pacificus *suggests these high evolutionary dynamics to be associated with copy number variations and positive selection.

**Conclusion:**

We could demonstrate functional integration of acquired cellulase genes into the nematode's biology as predicted by theory. Thus, functional assimilation, remarkable gene turnover and selection might represent key features of horizontal gene transfer events in nematodes.

## Background

Horizontal gene transfer (HGT), the movement of genes from one organism to another by mechanisms other than direct descent, is widespread in prokaryotes, but is thought to be rare among eukaryotes with sexual reproduction [1,2]. However, recent genome and expressed sequence tag (EST) projects in nematodes provide strong evidence for HGT from bacteria, fungi, amoebozoa or from endosymbionts in the plant parasites *Meloidogyne incognita *and *M. hapla*, fungivorous and plant-parasitic nematodes of the *Bursaphelenchus *group, the necromenic nematode *Pristionchus pacificus *and the filarial nematode *Brugia malayi *[3-7]. In particular, cell wall degrading enzymes of glycosyl hydrolase families (endo-1,4-beta-glucanases; cellulases), pectate lyases and xylanases were acquired by several of these nematodes. Cellulases (EC 3.2.1.4) can be found in 14 different glycosyl hydrolase families, of which only GHF5 and GHF45 were found in nematodes so far [8].

Surprisingly, phylogenetic reconstruction suggests that cellulase genes have been acquired multiple times independently in nematodes from distinct microbial donors [9]. Characterized cellulases from plant parasitic Tylenchida (plant sedentary endoparasitic nematodes such as *Meloidogyne*, *Heterodera *and *Globodera*) and migratory parasitic species (*Pratylenchus penetrans*) are from glycoside hydrolase family 5 (GHF5). They appear to have acquired a whole ancestral GHF5 gene cassette consisting of the catalytic domain and the carbohydrate-binding module 2 (CBM2) [10]. The intronless ancestral gene from putative bacterial donors later gained introns followed by intron shifts or losses in single gene copies. The pine wood nematode *Bursaphelenchus xylophilus*, which is part of the same clade as the cyst/root-knot nematodes above, but from a distinct grouping, acquired a different family of cellulases (GHF45) from fungi [3]. Genes obtained by HGT that are central to parasitic interaction, like pectin lyase or cellulase genes have undergone expansion by gene duplication after HGT in *Meloidogyne hapla*, *M. incognita *or *P. pacificus *[5-7,10].

Evolutionary theory makes a number of predictions for the successful acquisition of genes by HGT. Specifically, evolutionary theory predicts that the successful integration of HGT-acquired genes into the biology of the host requires gene activity and longevity [11], resulting in a number of testable, but yet unanswered questions, i.e. are genes acquired by HGT functional? How stable are these genes over evolutionary time scales and do they undergo normal or unusual turnover rates? Finally, are such genes under positive selection?

To study the evolutionary history, activity and longevity of genes acquired by HGT requires a well-established phylogenetic framework at the species and family level and a number of natural isolates for intra-species comparisons. Nematodes of the beetle-associated genus *Pristionchus *fulfill these requirements as more than 160 strains of worldwide origin are available for the model species *P. pacificus *and 25 species of the *Pristionchus *genus have been phylogenetically characterized [12,13]. Furthermore, molecular analysis places the genus *Pristionchus *well within the Diplogastridae family [14-16]. Building on this phylogenetic framework, we used, first, transcriptomics of seven *Pristionchus *species and three additional diplogastrid species to study the evolution of cellulase genes and, second, compared individual genes in 24 natural isolates of *P. pacificus*.

Here we show that species with cellulase genes in their transcriptome always exhibited cellulase activity. Cellulase genes show high turnover with significant birth and death rates. The comparison of 24 natural isolates of *P. pacificus *strains from around the world suggests these high evolutionary dynamics to be associated with copy number variations and positive selection. We conclude that functional assimilation, high gene turnover and selection might represent key features of HGT events in nematodes.

## Results

### Cellulase genes in diplogastrid transcriptomes

**Figure 1:**
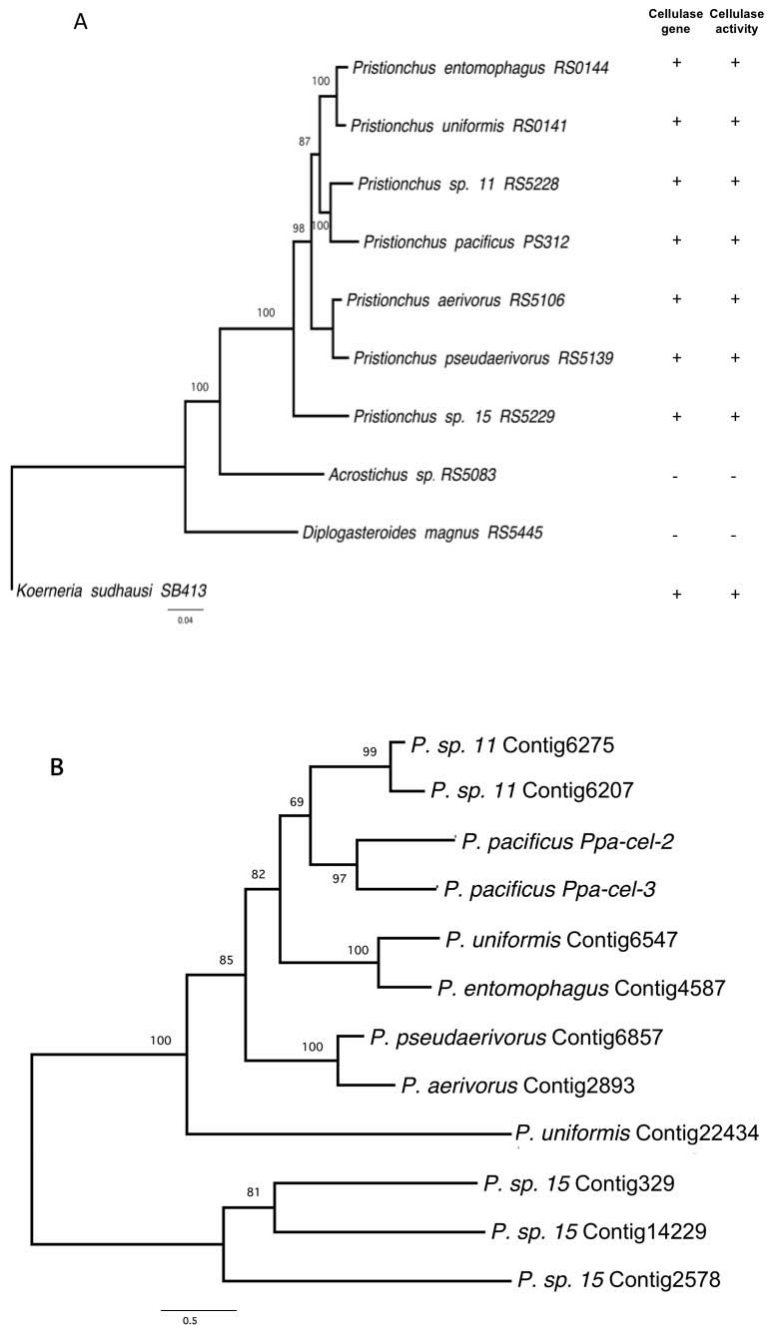
**Correlation between presence of cellulase genes and cellulase activity**. **(A) **Phylogenetic relationships and results of diplogastrid species tested for cellulase transcripts and activity. These 10 species were used in Roche 454 sequencing. The tree was reconstructed from 4,043 bp of concatenated ribosomal protein genes by heuristic search using the maximum likelihood criterion in the PAUP*4.0b10 software as described in Mayer et al. (2009) [11,12]. Numbers at nodes indicate bootstrap values. The presence of cellulase transcripts and observed cellulase activity in worm supernatants is indicated. The tree was rooted by *Koerneria sudhausi*. **(B) **Neighbour-joining tree of 246 bp of aligned EST contigs from Roche 454 transcriptomes encoding CBM49 domains of cellulases (see additional file 1). The best-fit substitution model (TrNef+G) has been selected by Modeltest 3.7 (Posada) corresponding to equal base frequencies, Nst = 6, substitution rates of A-C = 1, A-G = 1.1039, A-T = 1, C-G = 1, C-T = 1.7501, G-T = 1.0000; proportion of invariable sites I = 0; gamma distribution shape parameter α = 0.9033. Numbers at nodes indicate bootstrap values of 1,000 replications.

EST libraries of *P. pacificus*, six additional *Pristionchus *species, and three other diplogastrids (*Acrostichus *sp. RS5083, *Diplogasteroides magnus *RS5445 and *Koerneria sudhausi *SB413) were sequenced using the Roche 454-sequence technology platform (Figure 1A, see Materials and Methods for details) [17]. The datasets contained 16,000 to 28,000 assembled EST contigs and 42,000 to 148,000 singletons, each. Expressed gene contigs encompassing the carbohydrate-binding module (CBM) present at the C terminus of cellulases, were identified in all seven *Pristionchus *species and a near full-length cellulase transcript was found in *Koerneria sudhausi *(Figure 1A) [18]. In contrast, cellulase/CBM transcripts were not found in *D*. *magnus *and *Acrostichus *sp. RS5083 (Figure 1A). The CBM of the *Pristionchus *nematodes belonged to the CBM49 (pfam09478) family [19] and differed from that of plant-parasitic nematodes, which have similarity to the bacterial carbohydrate-binding module of family 2 (CBM2; pfam00553) [18]. The CBM49 domains of different *Pristionchus *species were all highly similar to each other (Figure 1B; additional file 1). In contrast, in the *K. sudhausi *cellulase a CBM domain could not be detected and the cellulase transcripts did not show sufficient sequence similarity to the *Pristionchus *cellulases to be included in the same multiple sequence alignments.

**Figure 2:**
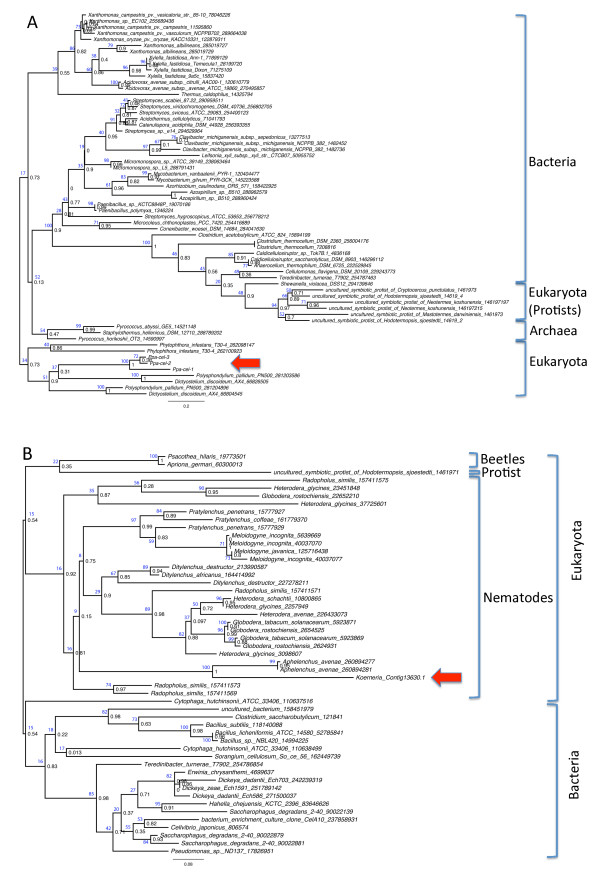
**Phylogenies of diplogastrid GHF5 cellulase proteins and the best non-diplogastrid matches**. The best protein matches from the GenBank database were identified and retrieved using BLAST Explorer and a phylogram was reconstructed using the Phylogeny.fr server by the PhyML (v3.0) algorithm. Paralogous genes were omitted from the analysis. The GenInfo Identifier (gi) numbers are given after the taxon names. Numbers in black print at nodes indicate approximate Likelihood-Ratio Test (aLRT) values, numbers in blue print indicate bootstrap support values. Red arrows indicate the query taxa. (A) Phylogenetic relationships of *P. pacificus *cellulases *Ppa-cel-1*, *Ppa-cel-2*, and *Ppa-cel-3 *to 63 best-matching proteins from GenBank using 109 amino acid positions after curation by the Gblocks algorithm. The initial tree was obtained by the BIONJ algorithm, the amino acid substitution model was set to WAG. The discrete gamma model was applied with 4 categories, a gamma shape parameter of 1.119, and proportion of invariant sites of 0.030. (B) Phylogenetic relationships of the *K. sudhausi *cellulase EST contig to 51 best-matching proteins from GenBank using 141 amino acid positions after curation by the Gblocks algorithm. The initial tree was obtained by the BIONJ algorithm, the amino acid substitution model was set to WAG. The discrete gamma model was applied with 4 categories, a gamma shape parameter of 1.294, and proportion of invariant sites of 0.097.

BLASTX searches of GenBank, Wormbase and UniRef90 databases with *Pristionchus *and *Koerneria *cellulase transcript sequences encompassing the full-length cellulase domain identified them as members of the glycosyl hydrolase family 5 (GHF5; pfam00150) (Figure 2) [6,20,21]. Different sets of closest matching sequences were found. The *Pristionchus *cellulase genes *Ppa-cel-2*, *Ppa-cel-3 *and *Ppa-cel-1*, the latter not detected in the sequenced transcriptome, were most closely related to a paralogous set of duplicated genes of the slime molds *Dictyostelium discoideum *and *Polysphondylium pallidum *(score: 264 bits, E-value: 4e-68), whereas *K. sudhausi *genes revealed high similarity to cellulase genes of the plant-parasitic nematode *Aphelenchus avenae *(score: 306 bits, E-value: 1e-81; Figure 2). These results of the similarity searches could be affected by skewed or insufficient sampling because matching homologues from related organisms are not yet in the databases, as has been argued by Mitreva et al. (2009) for GHF5 cellulase genes in plant-parasitic nematodes [22]. This explanation could hold for the *Koerneria *GHF5 cellulase gene, which appears to be in a monophyletic clade with plant-parasitic nematodes of the phylogenetic clade 12 [16]. However, the clade 9 nematode *Koerneria *is the only nematode in this clade - which encompasses Diplogastridae (*Pristionchus *and others) and Rhabditidae (*Caenorhabditis *and others) - to possess this GHF5 subclass, and no genes similar to GHF5 cellulases were detected in available EST datasets at GenBank from non-plant parasitic nematode clades or other species at intermediate phylogenetic positions (e. g., Rhabditida, 748,443 ESTs; Ascarida, 63,743 ESTs; Spirurida, 59,125 ESTs; Trichocephalida, 52,763 ESTs; Dorylaimida, 9,351 ESTs; Araelaimida, 2,591 ESTs).

In contrast, the *Pristionchus *cellulases belong to a GHF5 subclass that is distinct from those in plant-parasitic nematodes. Tracing the GHF5 genes of clade 9 and clade 12 nematodes and amoebozoans back to a common ancestor would require maintenance and sequence conservation of gene copies in genomes over extended evolutionary timescales and massive parallel losses of genes in most investigated nematodes and other phylogenetically related lineages of organisms during evolution. Hence, two independent HGT events, one from an amoebozoan or related microorganism to an ancestral *Pristionchus *species and a second from an unknown donor to *Koerneria *and *Aphelenchus*, appear to be the most likely scenario.

### Correlation of cellulase genes and cellulase activity

**Figure 3:**
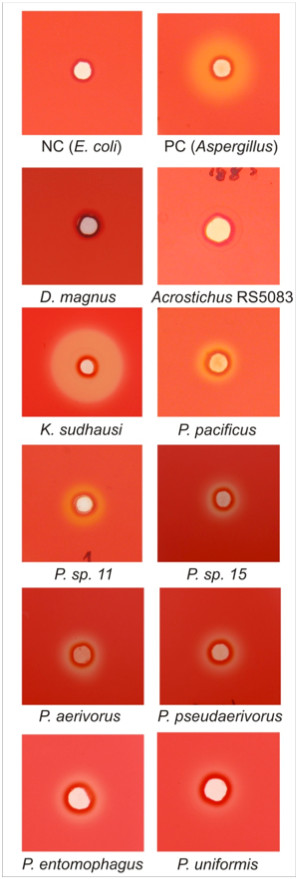
**Cellulase activity in diplogastrid nematodes**. The cellulase activity assays were performed on carboxymethylcellulose agar plates [3,23]. Clear halos were detected in the supernatants of *P. uniformis*, *P. entomophagus*, *P. aerivorus*, *P. pseudaerivorus*, *P*. sp. 11, *P*. sp. 15, and *K. sudhausi *mixed-stage cultures and in the positive control (*Aspergillus*). No halos were observed in the supernatants of mixed-stage cultures of *Acrostichus *sp. and *D. magnus *and the negative control (*Escherichia coli*).

Is the presence of cellulase genes in the EST sequence data correlated with cellulase activity? We tested the supernatant of mixed stage cultures of all ten species in a Congo red-polysaccharide interaction assay and found complete correlation with the transcriptome data [3,23]. All eight tested *Pristionchus *and *Koerneria *species that contain cellulase genes in their EST libraries had detectable cellulase activity in the assay (Figure 1A; Figure 3). In contrast, *D. magnus *and *Acrostichus *sp. RS5083 did neither show cellulase activity nor could we detect expression of cellulase genes in their transcriptomes (Figure 1A; Figure 3). Thus, species with cellulase genes in their transcriptome always exhibited cellulase activity, suggesting that functional assimilation of HGT acquired genes represents an important force during HGT events.

### Evolutionary dynamics of *Pristionchus *cellulase genes

To study the evolutionary dynamics and turnover rates of cellulase genes, we reconstructed the phylogenetic relationship of the 12 *Pristionchus *ESTs encoding CBM49 domains by the maximum likelihood criterion using the alignment shown in additional file 1.The resulting phylogram revealed two important findings (Figure 1B). First, the cellulase CBM49 gene phylogeny is highly congruent to the species phylogeny of *Pristionchus *(Figure 1A,B) [13]. This result indicates an ancestral acquisition of cellulase genes followed by maintenance of the gene through several speciation events and subsequent divergence by point mutations during the evolution of species. The only discrepancy is presented by the *P. uniformis *Contig22434.1, which appears to be a cellulase gene that was maintained only in *P. uniformis *but was lost in other *Pristionchus *species. Homologous genes in the *P. pacificus *genome with highest similarity to *P. uniformis *Contig22434.1 are *Ppa-cel-2 *and *Ppa-cel-3*. Second, the cellulase genes evolve dynamically by gene duplications as indicated by species-specific duplicates in *P. pacificus*, *P*. sp. 11 and *P*. sp. 15, respectively. In addition to lineage-specific gene duplications, putative gene deletions have occurred as exemplified by an ancestral CBM49 domain that is detectable in only one extant species, *P. uniformis*. These results suggest conspicuous evolutionary dynamics of cellulase genes in the genus *Pristionchus *with high gene birth and death rates.

Copy number variation among populations is well known from human evolution [24]. We wanted to know if the evolutionary dynamics of genes acquired by HGT also results in patterns of copy number variations. We selected three paralogous cellulase genes from the *P. pacificus *genome and compared them in 24 natural isolates of worldwide origin (Table 1). Two of the genes (*Ppa-cel-2 *and *Ppa-cel-3*) are expressed in the transcriptome and are detectable by mass spectroscopy in the proteome [6,25]. For comparison, the third gene (*Ppa-cel-1) *has been selected because it could be found as a complete copy in the genome and its transcription has been demonstrated, but at too low levels to be found in the transcriptome and proteome analyses [6,25]. The analyzed parts of the genes *Ppa-cel-1*, *Ppa-cel-2 *and *Ppa-cel-3 *encompass 3.0, 2.6 and 2.8 kb, respectively, with a total of 3.9 kb of exonic sequence (Table 2). While *Ppa-cel-1 *and *Ppa-cel-2 *were obtained from all 24 natural isolates, *Ppa-cel-3 *could only be detected in 16 out of the 24 *P. pacificus *strains (Table 1). The variability of *Ppa-cel-3 *among the strains is low, with ≤ 0.4% nucleotide differences in pair-wise comparisons of the whole gene and ≤ 0.3% nucleotide differences in exons. At the same time, this gene is highly similar to *Ppa-cel-2 *in transcript sequence and to a large part in intronic sequences with most of the splice sites being conserved between them. These results suggest that the *Ppa-cel-3 *arose by recent gene duplication from *Ppa-cel-2 *within the species *P. pacificus*. The time since its birth was too short for substantial sequence diversification and the gene itself has not yet spread through the whole population. Thus, the comparison of *Pristionchus *species and *P. pacificus *strains provides strong evidence for notable evolutionary turnover of cellulase genes by gene duplications and putative deletions.

**Table 1:** *Pristionchus pacificus *strains

Strain ID	Origin	Presence of *Ppa-cel-3*
PS312	USA	+

PS1843	USA	+

RS5200	India	-

RS5202	South Africa	+

RS5270	Bolivia	-

RS5275	Bolivia	-

RS5205	South Africa	+

RS5134	USA	+

RS5279	China	-

RS5171	Montenegro	+

RS5194	Japan	+

RS0106	Poland	+

JU482	Japan	+

JU723	China	-

JU138	Hawaii	+

JU150	Madagascar	-

RS5295	Switzerland	-

JU1089	Japan	-

RS5195	Japan	+

RS5282	Turkey	+

RS5302	Bali	+

RS5266	Bolivia	+

RS5296	USA	+

RS5297	USA	+

**Table 2:** *Pristionchus pacificus *cellulase gene data

Gene	Amplified segment	No. of exons/length	Likelihood for model M8 allowing positive selection^*^	Likelihood for model M8a not allowing positive selection^*^	Significance level^*^	synonymous substitutions	non-synonymous substitutions	overall average synonymous/non-synonymous substitutions
*Ppa-cel-1*	2,997 bp	13/1,177 bp	no positively selected sites		non-significant	0-1.8%	0-6.3%	4.6%/1.6%

*Ppa-cel-2*	2,642 bp	16/1,425 bp	-2705.39	-2820.06	0.001	0-2.2%	0-1.4%	1.3%/0.7%

*Ppa-cel-3*	2,837 bp	16/1,374 bp	no positively selected sites		non-significant	0-0.5%	0-0.3%	0.3%/0.1%

*Positive selection was determined using the Selecton server

### Evidence for positive selection of *Ppa-cel-2*

In addition to copy number variations, the evolutionary diversification of genes should result in intra-specific polymorphisms, which are either the result of non-adaptive or selective processes [26]. The *P. pacificus *collection of wild isolates of worldwide origin enables the inference of site-specific positive Darwinian and purifying selection. Site-specific positive Darwinian should result in a ratio of non-synonymous (K_a_) to synonymous (K_s_) substitutions higher than one at individual codons. We calculated the K_a_/K_s _ratio at each site of the cellulase proteins *Ppa*-CEL-1, *Ppa*-CEL-2 and *Ppa*-CEL-3 with the help of the Selecton server by using an empirical Bayesian method [27]. Sites with a K_a_/K_s _ratio ≥1 in *Ppa*-CEL-2 were identified. A likelihood ratio test was performed between the two models, M8 and M8a, implemented on this platform. M8 considers sites under purifying selection vs. a category of sites experiencing positive or neutral selection (ω_s _≥1) [28]. M8a, which is nested in the M8 model, is restricted to purifying and neutral selection only (ω_s _= 1) [29]. The test showed a significance level of 0.001 in support for the M8 model, which allows site-specific positive selection (Table 2).

In contrast, *Ppa*-CEL-1 and *Ppa*-CEL-3 did not show any evidence for positive selection, arguing that the evolutionary forces act on individual genes with high specificity. This is also observable when comparing the degree of polymorphisms among the three genes. The highly expressed *Ppa-cel-2 *gene displayed up to 2.1% nucleotide differences in pair-wise comparisons of the whole gene and 1.5% in exons. In contrast, the variation in *Ppa-cel-1 *was considerably higher with up to 5.7% and 6.0% nucleotide differences in the whole gene and exons, respectively. We speculate that neutral processes underlie this variability in *Ppa-cel-1*, but not in *Ppa-cel-2*. Taken together, the comparison of three cellulase genes in 24 natural isolates of *P. pacificus *indicates that different evolutionary forces act on these genes and provides evidence for positive selection acting on *Ppa-cel-2*.

### Evidence for intron gain after HGT

After a gene has been acquired by HGT it will assimilate to the new genomic environment, which may lead to changes in the size and number of spliceosomal introns [30]. Consistent with this, we found that the putative donor cellulase genes in the slime molds *D. discoideum *and *P. pallidum *contain only one or two small introns of 71 to 275 bp, whereas the *P. pacificus *cellulase genes *Ppa-cel-1*, *Ppa-cel-2*, and *Ppa-cel-3 *possess 13 to 16 introns, ranging from 14 to 452 bp (Table 2). This includes several extremely small introns of a length below 50 bp, which are typical for *P. pacificus *and other nematodes [6]. Assuming that the slime mold genes represent the ancestral state, these cellulase genes have gained up to 15 small spliceosomal introns. However, we want to note that in theory, it is also possible that slime molds might have lost ancestral introns.

## Discussion

We have shown that the diplogastrid nematode genera *Koerneria *and *Pristionchus *independently acquired cellulase genes by HGT from different putative donors. A detailed functional and genomic investigation in the phylogenetic framework of *Pristionchus *reveals that all features of evolutionary significance are fulfilled, including functional activity, longevity and the action of positive selection. Longevity of the transferred genes is indicated by their divergence from the donor through accumulated mutations. In addition, the resulting cellulase CBM49 phylogeny following HGT into *Pristionchus *is congruent to the host taxon phylogeny, i.e. the phylogenetic pattern of the transferred genes is largely identical to the phylogeny of the genus *Pristionchus *(Figure 1A,B). Thus, the original insertion of the cellulase gene occurred in an ancestral taxon and has been maintained over evolutionary time scales. During these processes, the cellulase gene became functionally integrated into the nematode's biology. Five notions support this. First, the cellulase genes are transcribed. Second, peptides corresponding to transcripts were identified by mass spectrometry [25]. Third, the nematodes secrete cellulases whose enzymatic activity can be demonstrated in functional assays with supernatant of mixed stage cultures. In nature, microorganismal and plant cellulose are potential targets for nematode cellulase activity, providing new potential food sources and fitness advantages for the nematode. Fourth, the cellulase genes gained introns in the host species, which are in phase with the functional open reading frame. The finding that the original open reading frames have not been disrupted by intron gain provides additional evidence for the functional assimilation of the acquired genes and for a selective advantage of active cellulase proteins in *P. pacificus*. Fifth, the cellulase genes display high intra-genus and intra-species turnover dynamics by birth-and-death processes, accompanied by selection for certain functional genes. In conclusion, we demonstrated the functional integration of acquired cellulase genes into the nematode's biology as predicted by theory. Functional assimilation, remarkable gene turnover and selection might represent key features of horizontal gene transfer events in nematodes. We speculate that HGT represents a major source for evolutionary innovation in nematodes and might have helped nematodes to invade the diversity of ecosystems, in which they can be found today, such as parasites of plants, animals, including humans.

## Conclusions

Predictions for successful horizontal gene transfer by evolutionary theory include, in particular, integration of the genes into the host's genome, functional activity, evolutionary longevity and positive selection of the genes. Supported by a robust phylogenetic framework we examined here these predictions for cellulase genes in seven species of the diplogastrid nematode *Pristionchus *and three other related diplogastrid genera. The following conclusions can be drawn. First, species with cellulase genes in their transcriptome always showed enzymatic cellulase activity demonstrating functional integration into the biology of the nematode. Second, different cellulase genes were acquired independently by the diplogastrid nematodes *Koerneria *and *Pristionchus*. Third, the considerable congruence of the phylogenetic profile of cellulase genes within the genus *Pristionchus *with the phylogeny of the species indicates an ancestral gene integration event into the genome and gene longevity. Forth, sequence comparisons of three selected cellulase genes in 24 natural isolates of the species *Pristionchus pacificus *show notable evolutionary dynamics with elevated birth and death rates, associated with copy number variations and putative positive selection for one of the cellulase genes. Thus, functional integration of acquired cellulase genes into the nematode's biology, as predicted by theory, could be demonstrated.

## Methods

### Expressed sequence tag (EST) library preparation and sequencing

Mixed stage cultures of inbred nematode strains derived from isogenic female lines were collected and washed with phosphate-buffered saline. The strains were: *Pristionchus pacificus *PS312; *P. aerivorus *RS5106; *P. pseudaerivorus *RS5139; *P. entomophagus *RS0144; *P. uniformis *RS0141; *P*. sp. 11 RS5228; *P*. sp. 15 RS5229; *Diplogasteroides magnus *RS5445; *Acrostichus *sp. RS5083; and *Koerneria sudhausi *SB413. Total RNA was prepared by the Triazol method (Invitrogen). Normalized cDNA libraries from the polyadenylated RNA fraction from nine of the mixed stage nematodes were constructed at the GSC at Washington University by standard procedures and sequenced on the Roche 454 FLX platform producing 320,000 to 540,000 reads. Median read length was ~ 250 bp for all libraries. The *K. sudhausi *library was sequenced on the Roche 454 Titanium platform resulting in 1.2 million reads. High quality Roche 454 sequencer reads were assembled with the EST version of PCAP.REP [31] to EST contigs and annotated by similarity searches using BLASTX against the non-redundant protein databases Uniref90 and GenBank. The complete ten species EST library dataset will be published elsewhere. Carbohydrate-binding modules (CBM) and cellulase families were identified and classified with the help of the NCBI conserved domain database (CDD) (http://www.ncbi.nlm.nih.gov/structure/cdd) and the Carbohydrate-Active EnZymes database (CAZy) server [8,32,33]. All cellulase genes identified in *Pristionchus *and *Koerneria *are available at Pristionchus.org under http://www.pristionchus.org/suppPlosMayer.html

### Multiple sequence alignments and phylogenetic tree reconstruction

Cellulase nucleotide sequences were aligned by Clustalx [34,35] and adjusted manually with the help of the Seqpup (v0.6f) software [36]. For phylogenetic tree reconstruction the optimal substitution model was selected by the Modeltest 3.7 software using the Akaike information criterion [37,38]. A phylogenetic tree was then obtained by the neighbour-joining algorithm with the help of the PAUP*4.0b10 software [39]. The distance measure was set to ML with likelihood settings corresponding to the model as determined by Modeltest. Trees were visualized using FigTree v.1.3.1 [40].

To find the closest homologs to the diplogastrid cellulase EST contigs the 100 best protein matches were identified by a similarity search in the non-redundant protein database (2010-05-18) at GenBank and retrieved using BLAST Explorer [41]. The dataset was pruned by removal of highly similar paralogous or allelic protein sequences. The minimum score of the sequences kept in the final dataset was 140 bits with an E-value of 9e-31 for *Pristionchus *cellulase EST contigs, and a score of 156 bits, E-value of 1e-36 for the *Koerneria sudhausi *EST contig. Phylogenetic analysis of the dataset was performed by the workflow of the Phylogeny.fr platform [42] comprising sequence alignment by the MUSCLE software (v3.7) [43], removal of ambiguous regions by Gblocks (v0.91b) [44] using the following parameters: minimum length of a block after gap cleaning: 10; no gap positions allowed in the final alignment; all segments with contiguous nonconserved positions bigger than 8 were rejected; minimum number of sequences for a flank position: 85%. The phylogenetic tree was reconstructed from the protein sequences using the maximum likelihood method implemented in the PhyML program (v3.0 aLRT) on the same platform [42,45]. WAG was used as substitution model with 4 substitution rate categories, number of invariant sites and gamma distribution parameter were estimated. The gamma shape parameter was estimated directly from the data (gamma = 1.119). Reliability of internal branches was assessed using the aLRT test (SH-Like) [46].

### Amplification of *Pristionchus pacificus *cellulase genes by polymerase chain reaction (PCR)

Cellulase genes from genomic DNA from 24 *P. pacificus *strains were amplified in four or five overlapping amplicons by PCR. The reactions were performed in 20 μl of 1× PCR buffer (GE HealthCare, Germany) containing 0.2 mM of each deoxynucleoside triphosphate, 0.5 μM of each primer, 40 ng of genomic DNA, and 1 unit of *Taq *DNA polymerase (GE HealthCare). The reactions were started by initial denaturation at 94°C for 5 min in a GeneAmp PCR System 9700 thermocycler (PE Applied Biosystems, Darmstadt, Germany), followed by 35 cycles of denaturation at 94°C for 30 sec, primer annealing at 55°C for 30 sec, and extension at 72°C for 2 min. A final incubation step at 72°C for 7 min concluded the reaction. The products were gel purified and directly sequenced by the PCR primers (additional file 2). The sequences were assembled using the SeqMan Pro (v8.0.2) software in the Lasergene 8 package (DNASTAR) and aligned to *P. pacificus *PS312 genomic and cDNA with the help of Seqpup (v0.6f) to deduce the positions of exons [6,36]. Protein sites under selection were identified in a codon-alignment of exon sequences using the Selecton server at http://selecton.tau.ac.il/[27].

### Cellulase Activity Assay

About 50,000 mixed stage worms per milliliter M9 medium were incubated on a shaking platform for 2 days at 20°C. The culture supernatants were sterile filtered and 40 μl were applied to holes in agar plates containing 0.5% carboxymethylcellulose sodium salt. After incubation for 24 h the plates were stained with 0.1% Congo red for 15 min and destained with 1 M NaCl for another 15 min. The assay was repeated at least 3 times for each nematode strain.

#### Accession codes

The nucleotide sequences reported here have been deposited at GenBank with the accession numbers HQ632018-HQ632092.

#### URLs

Pristionchus web server: http://www.pristionchus.org/suppPlosMayer.html. Selecton server: http://selecton.tau.ac.il/. Phylogeny.fr web service: http://www.phylogeny.fr/. Uniref90 database: http://www.ebi.ac.uk/uniref/. Genbank database: http://www.ncbi.nlm.nih.gov/. Carbohydrate-Active enZYmes Database (CAZy): http://www.cazy.org/.

## Abbreviations

CBM: Carbohydrate binding module; CNV: copy number variation; EST: expressed sequence tag; GHF: glycosyl hydrolase family; HGT: horizontal gene transfer; PCR: polymerase chain reaction

## Competing interests

The authors declare that they have no competing interests.

## **Authors' contributions**

WEM, CD and RJS conceived and designed the experiments. WEM, GB and LNS performed the experiments. WEM and CD analyzed the data. WEM, CD and RJS wrote the paper. RJS was the principal investigator and oversaw experimental design and analysis. All authors read and approved the final manuscript.

## Supplementary Material

Additional file 1 **Multiple sequence alignments of the *Pristionchus *CBM49 domains obtained from the transcriptomes**. **(A) **Nucleotide alignment. **(B) **Amino acid alignment. The alignments were created by Clustal × 2.0.11 [35]. Click here for file

Additional file 2 **Table PCR primers**. PCR primers used to amplify *P. pacificus *cellulase genes. Click here for file
