# Supplementary material for: Horizontal gene transfer of microbial cellulases into nematode genomes is associated with functional assimilation and gene turnover
Source: BMC Evol Biol. 2011 Jan 13;11:13. doi: 10.1186/1471-2148-11-13 (PMC3032686; doi:10.1186/1471-2148-11-13)
Supplement: Additional file 1 — Multiple sequence alignments of the Pristionchus CBM49 domains obtained from the transcriptomes. (A) Nucleotide alignment. (B) Amino acid alignment. The alignments were created by Clustal × 2.0.11 [35]. [file 1471-2148-11-13-S1.PDF]

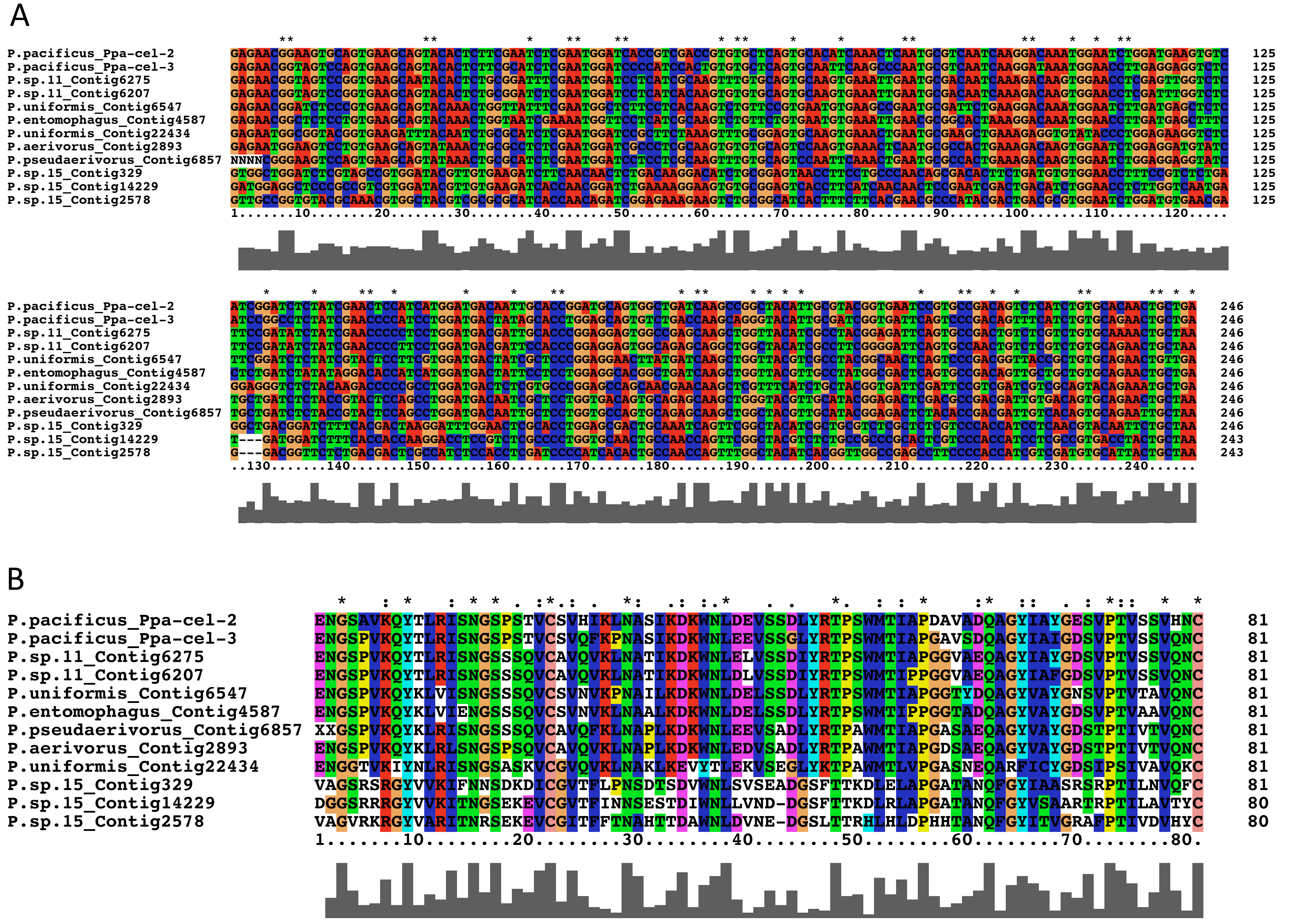
A
P.pacificus_Ppa-cel-2
P.pacificus_Ppa-cel-3
P.sp.11_Contig6275
P.sp.11_Contig6207
P.uniformis_Contig6547
P.entomophagus_Contig4587
P.uniformis_Contig22434
P.aerivorus_Contig2893
P.pseudaerivorus_Contig6857
P.sp.15_Contig329
P.sp.15_Contig14229
P.sp.15_Contig2578
125
246
243
B
P.pacificus_Ppa-cel-2
P.pacificus_Ppa-cel-3
P.sp.11_Contig6275
P.sp.11_Contig6207
P.uniformis_Contig6547
P.entomophagus_Contig4587
P.pseudaerivorus_Contig6857
P.aerivorus_Contig2893
P.uniformis_Contig22434
P.sp.15_Contig329
P.sp.15_Contig14229
P.sp.15_Contig2578
81
80
