# Supplementary material for: Horizontal gene transfer of microbial cellulases into nematode genomes is associated with functional assimilation and gene turnover
Source: BMC Evol Biol. 2011 Jan 13;11:13. doi: 10.1186/1471-2148-11-13 (PMC3032686; doi:10.1186/1471-2148-11-13)
Supplement: Additional file 2 — Table PCR primers. PCR primers used to amplify P. pacificus cellulase genes. [file 1471-2148-11-13-S2.DOC]

Additional file 2 – PCR primers.

___________________________________________________________________

Name Oligonucleotide sequence Orientation Gene

___________________________________________________________________

WM23645 TCGAATCCGTATAATTTTAAGCCA sense *Ppa-cel-1*

WM23646 ATACAGGTAGAGAACACGCACGAA antisense *Ppa-cel-1*

WM23647 AGAAGAAGAAATGGTGAAGAGTAG sense *Ppa-cel-1*

WM23648 GTGGTAACCTAAATCCGAGAATGA antisense *Ppa-cel-1*

WM23649 GTAGCAATGAGGCGAGTAGACGAC sense *Ppa-cel-1*

WM23650 TGAGATTTACAGTGCCGCCGAACG antisense *Ppa-cel-1*

WM23651 TTATCAAATAGGACTCACCACAGC sense *Ppa-cel-1*

WM23652 CGATGGAGATGGTCAGGTAAGAAC antisense *Ppa-cel-1*

WM23653 TGTCTGGCTGAGTAATCCGTTTCT sense *Ppa-cel-1*

WM23654 CTTCTGGGCCTTCTTGCTTTCGTT antisense *Ppa-cel-1*

WM23336 AGTTACGACTTCTGTTGCTCTTACC sense *Ppa-cel-2*

WM23337 ATGAGACTGTCGGCACGGATTCACC antisense *Ppa-cel-2*

WM23338 AGTTACGACTTCTGTTGCTCTTACC sense *Ppa-cel-2*

WM23339 CGTAGCAGTGAGGTGAGTAGACAAC antisense *Ppa-cel-2*

WM23340 TTCGTTTCTCGGTCTCTTCTTCGTA sense *Ppa-cel-2*

WM23341 ATTGGAGCCGAGGTGGTGATAGTTG antisense *Ppa-cel-2*

WM23342 AAGTGGGAGTGAAAGTGACGGATGG sense *Ppa-cel-2*

WM23343 CAGATGAGACTGTCGGCACGGATTC antisense *Ppa-cel-2*

WM23372 GGCCATAACGTTTCTCATCCACTC sense *Ppa-cel-3*

WM23373 TGCACTTGCCTATCGCTGTATCTC antisense *Ppa-cel-3*

WM23374 AAGTCTACCAATCACAACCAGCAC sense *Ppa-cel-3*

WM23375 TCCACCCCATTCACCCACTACCAC antisense *Ppa-cel-3*

WM23376 GTAAATCAGACAGGTTCGCAGTAT sense *Ppa-cel-3*

WM23377 TGCCCACTCGCCTGCCATTCATTA antisense *Ppa-cel-3*

WM23378 AGTGGGAGGAGGGCAGGTGACGAC sense *Ppa-cel-3*

WM23379 ATGAAACTGTCGGGACTGAATCAC antisense *Ppa-cel-3*

___________________________________________________________________
